# Entropy and Ergodicity of Boole-Type Transformations

**DOI:** 10.3390/e23111405

**Published:** 2021-10-26

**Authors:** Denis Blackmore, Alexander A. Balinsky, Radoslaw Kycia, Anatolij K. Prykarpatski

**Affiliations:** 1 Department of Mathematical Sciences and CAMS, New Jersey Institute of Technology, Newark, NJ 07102, USA; 2 Mathematics Institute at the Cardiff University, Cardiff CF24 4AG, UK; BalinskyA@cardiff.ac.uk; 3 Faculty of Physics, Mathematics and Computer Science, Cracow University of Technology, 31-155 Kraków, Poland; kycia.radoslaw@gmail.com (R.K.); pryk.anat@cybergal.com (A.K.P.)

**Keywords:** discrete transformations, invariant measure, ergodicity, entropy, Bernoulli type transformations, Boole-type transformations, fibered multidimensional mappings, induced transformations

## Abstract

We review some analytic, measure-theoretic and topological techniques for studying ergodicity and entropy of discrete dynamical systems, with a focus on Boole-type transformations and their generalizations. In particular, we present a new proof of the ergodicity of the 1-dimensional Boole map and prove that a certain 2-dimensional generalization is also ergodic. Moreover, we compute and demonstrate the equivalence of metric and topological entropies of the 1-dimensional Boole map employing “compactified”representations and well-known formulas. Several examples are included to illustrate the results. We also introduce new multidimensional Boole-type transformations invariant with respect to higher dimensional Lebesgue measures and investigate their ergodicity and metric and topological entropies.

## 1. Introduction

With its origins going back several centuries, analysis of discrete dynamical systems has become an increasingly central methodology for many mathematical problems related to a wide range of applications in modern science and engineering. Our focus on the ergodic, topological and metric entropy aspects of discrete dynamical systems, especially those of Boole-type, [1,2,3,4,5,6,7,8,9,10,11,12,13,14] is apt owing to the importance and enduring interest in these areas of mathematical inquiry [13,14,15,16,17,18,19,20,21,22,23,24,25,26,27,28,29]. Ergodicity and entropy are properties of considerable interest in many fields; especially in statistical mechanics and thermodynamics [15,19,21,30,31,32,33], discrete mathematics, numerical analysis, chaos theory, statistics and probability theory as well as in electrical and electronic engineering.

The measurable dynamical systems oriented concept of metric entropy (K-S entropy) was introduced by A. N. Kolmogorov [34] as a useful invariant that could be used, for example, to solve the problem of showing the nonequivalence of the Bernoulli 2- and 3-shifts. It was also observed that the metric entropy is related to the exponential growth of distinguishable orbits, which in turn has a certain communication interpretation following from the fact that information theory models can be reformulated as Bernoulli schemes (cf. [35]). As a matter of fact, in that same year, C. Shannon independently introduced his theory of transmission along noisy channels and defined his entropy as a measure of information content. Interestingly, but not really surprising in view of the communication interpretation, Shannon entropy shares many features with metric entropy.

Topological entropy (AKM entropy) was introduced in 1965 by Adler, Konheim and McAndrew [36], and can be viewed as a means of providing an analog of K-S entropy for dynamical systems on topological spaces, independent of any measure-theoretic structure. All three versions of entropy are connected in a variety of aspects, as indicated, for example, in [35]. One of the more profound and rigorous connections is the variational principle stating that the topological entropy is the supremum of the metric entropy over all invariant Borel probability measures on the phase space of the dynamical system, as shown in [37]; a result that, for instance, is useful in studying weighted metric entropies (see, e.g., [38]). Ergodicity is another property of dynamical systems that is useful in establishing relationships between K-S and AKM entropy, such as in Theorem 2 of [35], and is a companion theme of this investigation. Moreover, ergodicity enables the derivation of precise formulas for K-S entropy for certain kinds of discrete dynamical systems, which can be used to determine the generally much harder to compute topological entropy when the two entropies can be shown to be equal. As we shall show, these ideas come into play in significant ways when dealing with Boole-type mappings.

It should be noted that the finiteness of the phase space, say *X*, is required in the classical definitions of dynamical systems entropies: the finiteness of the measure of *X*—so that it can be re-scaled to a probability measure—in the case of metric entropy and finiteness of open subcoverings of *X* guaranteed by the compactness of the phase space for topological entropy. Unfortunately, the Boole maps investigated in the sequel are invariant for Lebesgue measures that are only σ-finite on noncompact Euclidean spaces. So, for example, the nice Krengel–Rokhlin formulas for ergodic maps cannot be applied directly to compute the metric entropy nor can the fundamental Adler–Konheim–McAndrew definition of topological entropy be employed. Fortunately, methods, which essentially involve some sort of compactification, have been devised to deal with σ-finite invariant measures and noncompact spaces such as in [39,40,41,42,43,44,45]. We note that the extensions of metric entropy and similar invariants to the nonsingular domain, include Krengel’s entropy of conservative measure-preserving maps and its extension to nonsingular maps, Parry’s entropy and his nonsingular version of the Shannon–McMillan–Breiman theorem, and critical dimension by Mortiss and Dooley. Unfortunately, these invariants are usually less informative than their classical counterparts and more difficult to compute.

Our investigation is organized as follows. In Section 2, we describe some elements of metric entropy that are closely linked to ergodicity such as the Shannon–McMillan–Breiman theorem and the Krengel–Rokhlin formula and recall some useful results from classical measure theory. In addition, we define the notion of smooth fibered mapping for which metric entropy formulas can often be found, and illustrate applications of the elements and results to some examples; namely, the period-doubling measurable dynamical system and the classical continued fraction system. Next, in Section 3, we employ the results from Section 2 together with some limiting, compactification-type techniques and an entropy equivalence theorem to compute the K-S and AKM entropy of the (1-dimensional, Lebesgue measure invariant) Boole map dynamical system. Moreover, we present a new proof of the ergodicity of this Boole system, which is shorter than the original of Adler and Weiss [46], and compute the metric entropies of some 1-dimensional variations of the classical Boole system. Next, we introduce a class of higher-dimensional generalizations of the classical Boole system in Section 4 and prove the ergodicity of a 2-dimensional version. We also discuss the very useful concept of an induced transformation and some of its characterizations. Finally, we summarize the results obtained and indicate a few interesting directions for related future research in Section 5.

## 2. Metric Entropy, Ergodicity and Bernoulli Shifts

It is well known that metric entropy [17,26,47,48] for a measure preserving mapping f:X→X on the probability space (X;B,μ), is invariant under appropriately defined isomorphisms of X, so it is a useful numerical invariant of measurable dynamical systems. The K-S entropy $h_{\mu }\left(f\right)\in R_{+}$ of a map f:X→X is defined as follows: the entropy of a countable measurable partition $\xi :=\{A_{j}\in X:X:=$$\underset{j\in N}{∐}A_{j},A_{j}\cap A_{i}=⌀,i,j\in N\}$ of the probability space (X,B,μ) is defined [35,47,48,49] by means of the classical Gibbs expression:

(1) $$H_{\mu }\left(\xi \right):=-\sum_{j\in N}\mu \left(A_{j}\right)\ln\mu \left(A_{j}\right).$$

Then one can naturally construct an induced infinite sequence of refinements of the partitions of the form

(2) $$\begin{array}{c} \xi_{n}\left(f\right)=\vee_{i=0}^{n-1}f^{-i}\xi :=\{A_{k_{0}}\cap f^{-1}A_{k_{1}}\cap f^{-2}A_{k_{2}}\cap \cdots \\ \cap f^{-n+1}A_{k_{n-1}}:A_{k_{j}}\in \xi ,k_{j}\in N,j=\bar{0,n-1}\} \end{array}$$

for arbitrary n∈N and, whereupon (1), the metric entropy $h_{\mu }\left(f\right)\in R_{+}\;$ is defined as

(3) $$h_{\mu }\left(f\right):=\underset{\;H_{\mu }\left(\xi \right)<\infty }{\sup}\lim_{n\to \infty }n^{-1}H_{\mu }\left(\xi_{n}\left(f\right)\right).$$

There is an analogous definition of the topological entropy $h_{T}\left(f\right)$ for a continuous map on a topological space (X;T) described in sources such as [35,36] for compact spaces and in [44,45] for noncompact spaces. Computing metric entropy tends to be quite a bit easier than topological entropy. However, calculation $h_{\mu }\left(f\right)$ via definition (3) is generally very difficult, which naturally led researchers to find simpler methods for determining the metric entropy. Ergodicity, which is an important property in its own right, turns out to be a key to substantial simplifications in computing K-S entropy, such as in the the following result of Shannon–McMillan–Breiman [17,48,49]:

**Theorem** **1.** *Let a measurable mapping f:X→X on the probability space (X;B,μ) be ergodic and let ξ be a countably infinite generating partition of X, for which $H_{\mu }\left(\xi \right)<\infty .$ Then for μ-almost every x∈X,*

(4) $$h_{\mu }\left(f\right)=-\lim_{n\to \infty }n^{-1}\ln\mu \left(A_{n}\left(f;x\right)\right),$$

*where sets*$A_{n}\left(f;x\right)\in \xi_{n}\left(f\right),n\in N,$*are chosen so that*$x\in A_{n}\left(f;x\right),n\in N$.

The expression (4) simplifies the calculation of metric entropy for ergodic systems, but a fairly high level of difficulty remains. A further, very substantial simplification under the added assumptions of differentiability and dilation, was obtained by Krengel [50] and Rokhlin [51,52]; namely, if the measurable dynamical system (X;B,μ,f) is ergodic, *X* is a metric space and *f* is a *differentiable dilation* in the sense that the Radon–Nikodym derivative $f_{\mu }^{\prime }\left(x\right):=$dμ∘f(x)/dμ(x) at all points x∈X of the shifted measure μ∘f with respect to the probability measure μ is measurable and satisfies the dilation condition $\inf_{x\in X}f_{\mu }^{\prime }\left(x\right)>1$, then we have the simple formula

(5) $$h_{\mu }\left(f\right)=\int_{X}\ln f_{\mu }^{\prime }\left(x\right)d\mu \left(x\right),$$

strongly based on the Lyapunov exponents [48] of the mapping f:X→X on the probability space (X;B,μ).

We note that the dilation condition plays an important role in the existence of an invariant measure μ on *X* for f:X→X as shown in [47,48,49,53,54,55,56,57] for a wide class of measurable spaces.

In particular, let a smooth ($C^{1}$) measure preserving mapping f:X→X, defined on a metric space *X* be dilating [47,48,55,56], so that $\inf_{x\in X}f_{\mu }^{\prime }\left(x\right)>1$. Furthermore, let the finite generating partition $\xi :=\{A_{j}\in X:j=\bar{1,k}\},k\in N,$ be such that sets $f\left(A_{j}\right)\subset X,j=\bar{1,k},$ are measurable and the reduced (restriction) mappings $f_{j}:A_{j}\to f\left(A_{j}\right),j=\bar{1,k},$ are invertible and measurable. Then extend the inverse mappings $f_{j}^{-1}:f\left(A_{j}\right)\to A_{j},j=\bar{1,k},$ arbitrarily, but measurably, to the whole space X. Then, the entropy of the probabilistic measure invariant mapping f:X→X can be calculated, owing to the Gibbs Formula (1), as

(6) $$h_{\mu }\left(f\right):=\underset{\xi }{\sup}H\left(\xi |f^{-1}B\right)=-\underset{\xi }{\sup}\sum_{j=\bar{1,k}}\int_{X}\chi_{A_{j}}\left(x\right)\ln E\left(\chi_{A_{j}}|f^{-1}B\right)\left(x\right)d\mu \left(x\right),$$

where mappings $E\left(\chi_{A_{j}}|f^{-1}B\right):X\to R,j=\bar{1,k},$ denote the corresponding conditional expectations of the characteristic functions $\chi_{A_{j}}\left(\circ \right):X\to R,j=\bar{1,k},$ with respect to the σ-algebra $f^{-1}B.$ Taking into account that for any function $F\in L_{1}\left(X;R\right)$ one has for any x∈X the expansion

(7) $$F\left(x\right)=\sum_{j=\bar{1,n}}\chi_{A_{j}}\left(x|B\right)F\circ f_{j}^{-1}\circ f\left(x\right)\;$$

with respect to the Borel σ-algebra B, from the corresponding definition of the integrated conditional expectation value

(8) $$\sum_{j=\bar{1,n}}\int_{X}F\circ f\left(x\right)E\left(\chi_{A_{j}}|f^{-1}B\right)\left(x\right)d\mu \left(x\right)=\sum_{j=\bar{1,n}}\int_{A_{j}}F\circ f\left(x\right)d\mu \left(x\right),\;\;$$

it follows from (6) and (8) that

(9) $$\begin{array}{c} \int_{X}\;F\circ f\left(x\right)E\left(\chi_{A_{j}}\circ f|f^{-1}B\right)\left(x\right)d\mu \left(x\right)=\;\int_{A_{j}}F\circ f\left(x\right)d\mu \left(x\right)\\ =\;\int_{A_{j}}F\circ f\left(x\right){\left(\frac{d\mu \circ f(x)}{d\mu (x)}\right)}^{-1}d\mu \circ f\left(x\right)={\left.\int_{\varphi A_{j}}F\left(y\right)\frac{\chi_{f(A_{j})}\left(y\right)\;}{f_{\mu }^{\prime }\circ f_{j}^{-1}\left(y\right)}d\mu \left(y\right)\right|}_{\begin{array}{c} y\to f(x)\\ (measure\\ invariance) \end{array}}\\ =\int_{A_{j}}F\circ f\left(x\right)\frac{\chi_{f(A_{j})}\circ f\left(x\right)\;}{f_{\mu }^{\prime }\circ f_{j}^{-1}\circ f\left(x\right)}d\mu \left(x\right)=\int_{X}F\circ f\left(x\right)\frac{\chi_{f(A_{j})}\circ f\left(x\right)}{f_{\mu }^{\prime }\left(x\right)}d\mu \left(x\right), \end{array}$$

or equivalently,

(10) $$E\left(\chi_{A_{j}}|f^{-1}B\right)\left(x\right)=\frac{\chi_{f(A_{j})}\circ f\left(x\right)}{f_{\mu }^{\prime }\left(x\right)}$$

for all x∈X and $\;j=\bar{1,k}.$ Now, having substituted the conditional expectation functions (10) into (6), one finally obtains

(11) $$\begin{array}{cc} h_{\mu }\left(f\right) & =-\underset{\xi }{\sup}\sum_{j=\bar{1,n}}\int_{A_{j}}\chi_{A_{j}}\left(x\right)\ln E\left(\chi_{A_{j}}|f^{-1}B\right)\left(x\right)d\mu \left(x\right)=\\ \; & =\sum_{j=\bar{1,n}}\int_{A_{j}}\ln f_{\mu }^{\prime }\left(x\right)d\mu \left(x\right)=\int_{X}\ln f_{\mu }^{\prime }\left(x\right)d\mu \left(x\right)\; \end{array}$$

with respect to the covering finite generating partition ξ, which coincides with (5). If the measurable metric space (X;B,μ) is finite dimensional and the probabilistic measure dμ(x)=dλ(x),x∈X (the normalized Lebesgue measure on X), then the Radon–Nikodym derivative $f_{\mu }^{\prime }\left(x\right)=\left|J_{f}\left(x\right)\right|$ at point x∈X is the absolute value of the usual Jacobian of the differentiable mapping f:X→X, and then the Krengel–Rokhlin entropy expression (5) becomes

(12) $$h_{\mu }\left(f\right)=\int_{X}\ln\det\left|J_{f}\left(x\right)\right|d\lambda \left(x\right).$$

Using the Shannon–McMillan–Breiman expression (4), Formula (5) was also proved by Yuri [57] for multidimensional mappings with finite range structure subject to the corresponding partitions $\xi_{n}\left(\varphi \right),n\in N,$ induced by a fixed generating partition $\xi \in 2^{X},X\subset R^{n}.$ Among this class of mappings there are so called “fibered” mappings f:X→X satisfying the following conditions [5,14,26,57,58,59,60,61,62]:

(a) there is an invariant Lebesgue equivalent probability measure $\mu :B\to R_{+},$ for which there exist positive constants $c_{1},c_{2}\in R_{+},$ such that $c_{1}\lambda \left(E\right)\leq \mu \left(E\right)\leq c_{2}\lambda \left(E\right)$ for every Borel set E⊂X;
(b) there is a finite or countably infinite digit set $\;D_{j},j=\bar{1,N};$
(c) there is a mapping $k:X\to D,D:=\times_{j=\bar{1,N}}D_{j},$ such that the sets $X_{i}:=k^{-1}\left\{i\right\}=\left\{x\in X:k\left(x\right)=i\right\},i\in D,$ are measurable and form a partition ξ(X) of the space X, that is, ${⊔}_{i\in D}X_{i}=X;$
(d) the restrictions ${f|}_{X_{i}}:X_{i}\to X,i\in D,$ are injective and smooth.

Then one can see [5,51,57] that under the additional conditions imposed on the map *f*:X→X:

(13) $$\sum_{i\in D}\lambda \left(X_{i}\right)<\infty ;\vee_{n\in N}f^{-1}\left(\xi \right)X))=\tau \in X,$$

for some point τ∈X, it appears to be ergodic and equivalent to the weak Bernoulli shift mapping $\;T_{f}:D^{\infty }\to D^{\infty },$ where

(14) $$T_{f}:\left(k_{1},k_{2},k_{3},\cdots k_{n},\cdots \right)\to \left(k_{2},k_{3},\cdots k_{n},\cdots \right)\;$$

with respect to the isomorphism $\psi :X∋x\to \left(k_{2},k_{3},\cdots k_{n},\cdots \right)\in D^{\infty },$

(15) $$X_{n}\left(x\right):=X\left(k_{1},k_{2},k_{3},\cdots ,k_{n};x\right)⟺\left(k_{1},k_{2},k_{3},\cdots k_{n},\cdots \right),$$

determined for the admissible rank-*n* cylinder sets $X_{n}\left(k_{1},k_{2},k_{3},\cdots ,k_{n}\right)\subset X,n\in N,$ for which

(16) $$X_{n}\left(k_{1},k_{2},k_{3},\cdots ,k_{n}\right):=\cap_{j=\bar{1,n}}X_{k_{j}}\left(x\right),$$

and on which we shall not dwell.

In many concrete cases the ergodicity of a mapping f:X→X can be proved more effectively using standard measure theoretical calculations. In particular, using the construction above one can employ a slightly modified approach to [14,48,49] for proving ergodicity, making use of the following two classical measure theory [63,64] lemmas.

**Lemma** **1.** (Hahn–Caratheodory–Kolmogorov extension theorem)*Let A be an algebra of subsets of X and B(A) denote the σ-algebra generated by A. Suppose that a mapping μ:A→[0,1] satisfies the conditions:*

*(a) μ(⌀)=0; (b) if $A_{n}\in A,n\in N,$ are pair wise disjoint and if ${⊔}_{n\in N}A_{n}\in A,$ then $\mu \left({⊔}_{n\in N}A_{n}\right)=\sum_{n\in N}\mu \left(A_{n}\right).$ Then there is a unique probability measure μ:B(A)→[0,1], which is an extension of the mapping μ:A→[0,1].*

**Lemma** **2.** *Let (X,B,μ) be a probability space and suppose that A⊂B is an algebra that generates B, that is B=B(A). Suppose there exists C>0 such that for a fixed B∈B one has*

(17) μ(B)μ(I)≤Cμ(B∩I)

*for all I∈A. Then the measure $\mu \left(B\right)\mu \left(\bar{B}\right)=$0, where $\bar{B}:=X\setminus B\in B$ denotes the complement of the set B∈B.*

Owing to the weak equivalence of the above “fibered” mappings $f:X\to X,X\subset R^{n},$ to the Bernoulli shifts [47,48,57] (14), one can state the following important result.

**Theorem** **2.** *Let the cylinder sets of a smooth “fibered” mappings f:X→X with finite range structure satisfy the conditions of Lemma 2 for the case of the Lebesgue measure λ on X. Then, if the invariant measure μ is absolutely equivalent to the Lebesgue measure λ on X, the mapping f:X→X is ergodic.*

**Example** **1.** *One of the simplest examples is given by the doubling (2x mod1) map*

(18) f:[0,1)∋x→{2x}∈[0,1),

*where $k:\left[0,1\right)∋x\to \left\lfloor 2x\right\rfloor \in \left\{0,1\right\}:=D.$*

The doubling map is ergodic [49] with respect to the finite Lebesgue measure dλ(x)=dx,x∈[0,1), and allows the generating partition $\xi =\{X_{0}=\left[0,1/2\right),X_{1}=\left[1/2,1\right)\},$$X_{0}⊔X_{1}=\left[0,1\right)=X.$ Its entropy is easily calculated using the Krengel–Rokhlin Formula (5), as

(19) $$h_{\mu }\left(f\right)=\int_{0}^{1}\ln\left|f^{\prime }\left(x\right)\right|dx=\int_{0}^{1}\ln2dx=\ln2,$$

which is a well-known [47,48] result.

As for the ergodicity of (18), it can be easily proved by representing any number x∈[0,1) as a binary expansion

(20) $$x:=\left(\cdot x_{0}x_{1}x_{2}\cdots x_{n}\cdots \right)=\sum_{j\in Z_{+}}x_{j}2^{-(j+1)},$$

where x∈{0,1}=D. Denoting for convenience the set of all such expansions by Y=$\left\{\left(\cdot x_{0}x_{1}x_{2}\cdots x_{n}\cdots \right):x_{j}\in \left\{0,1\right\}\right\}≃{\left\{0,1\right\}}^{Z_{+}}$ makes it clear that the mapping (18) is equivalent to the left Bernoulli shift

(21) $$T_{f}\left(\cdot x_{0}x_{1}x_{2}\cdots x_{n}\cdots \right)=\left(\cdot x_{1}x_{2}\cdots x_{n}\cdots \right)$$

for any element $(\cdot x_{0}x_{1}x_{2}\cdots x_{n}\cdots )\in Y.$ Now one can introduce so called dyadic intervals or cylinder sets

(22) $$I\left(k_{0},k_{1},\cdots ,k_{n-1}\right)=\left\{x\in \left[0,1\right):x_{j}=k_{j},j=\bar{1,n-1}\right\},$$

where, for instance, I(0)=[0,1/2),I(1)=[1/2,1),I(0,0)=[0,1/4), and I(0,1)=[1.4,1/2). If A denotes the algebra of finite unions of such cylinders, it generates the usual Borel σ-algebra B of the interval [0,1). Moreover, if one takes two separate points *x*≠ y∈[0,1), their expansions are different at some place $n\in Z_{+}$ so these numbers belong to different disjoint cylinders. Now define the following inverse to (18) mappings $\sigma_{0}:\left[0,1\right)\to \left[0,1/2\right)$, and $\sigma_{1}:\left[0,1\right)\to \left[1/2,1\right),$ where

(23) $$\begin{array}{cc} \sigma_{0}\left(x\right) & =\left\{\begin{array}{ccc} x/2, & if & x\in [0,1/2) \end{array}\right.,\\ \sigma_{1}\left(x\right) & =\left\{\begin{array}{ccc} (1+x)/2, & if & x\in [1/2,1), \end{array}\right. \end{array}$$

where $\varphi \circ \sigma_{j}\left(x\right)=x,j=\bar{0,1},$ for any x∈[0,1) and whose actions on elements of the set *Y* are the corresponding right shifts:

(24) $$\begin{array}{cc} \sigma_{0}\left(\cdot x_{0}x_{1}x_{2}\cdots x_{n}\cdots \right) & =(\cdot 0x_{0}x_{1}x_{2}\cdots x_{n}\cdots ),\\ \sigma_{1}\left(\cdot x_{0}x_{1}x_{2}\cdots x_{n}\cdots \right) & =(\cdot 1x_{0}x_{1}x_{2}\cdots x_{n}\cdots ), \end{array}$$

It follows from definitions of cylinder sets (22) and actions (24) that

(25) $$I_{n}:=I\left(k_{0},k_{1},\cdots ,k_{n}\right)=\sigma_{k_{0}}\circ \sigma_{k_{1}}\circ \sigma_{k_{2}}\circ \cdots \circ \sigma_{k_{n}}\left(\left[0,1\right)\right),$$

with a readily calculated Lebesgue measure

(26) $$\lambda \left(I_{n}\right)=2^{-(n+1)}\sum_{j\in Z_{+}}2^{-j}=2^{-n}$$

for any n∈N.

We are now in a position to apply Lemmas 1 and 2. Let a measurable set B⊂[0,1) be *f*-invariant and calculate the Lebesgue measure

(27) $$\begin{array}{c} \lambda \left(B\cap I_{n}\right)=\int_{\left[0,1\right)}\chi_{B\cap I_{n}}\left(x\right)dx=\int_{\left[0,1\right)}\chi_{B}\left(z\right)\chi_{I_{n}}\left(x\right)dx=\int_{I_{n}}\chi_{B}\left(x\right)dx\\ =\int_{\left[0,1\right)}\chi_{B}\left(\sigma_{k_{0}}\circ \sigma_{k_{1}}\circ \sigma_{k_{2}}\circ \cdots \circ \sigma_{k_{n}}\left(x\right)\right)d\left(\sigma_{k_{0}}\circ \sigma_{k_{1}}\circ \sigma_{k_{2}}\circ \cdots \circ \sigma_{k_{n}}\left(x\right)\right)\\ =\int_{\left[0,1\right)}\chi_{{\tilde{\varphi }}^{-n}B}\left(\sigma_{k_{0}}\circ \sigma_{k_{1}}\circ \sigma_{k_{2}}\circ \cdots \circ \sigma_{k_{n}}\left(x\right)\right)d\left(\sigma_{k_{0}}\circ \sigma_{k_{1}}\circ \sigma_{k_{2}}\circ \cdots \circ \sigma_{k_{n}}\left(x\right)\right)\\ =\int_{\left[0,1\right)}\chi_{B}\left({\tilde{\varphi }}^{n}\circ \sigma_{k_{0}}\circ \sigma_{k_{1}}\circ \sigma_{k_{2}}\circ \cdots \circ \sigma_{k_{n}}\left(x\right)\right)d\left(\sigma_{k_{0}}\circ \sigma_{k_{1}}\circ \sigma_{k_{2}}\circ \cdots \circ \sigma_{k_{n}}\left(x\right)\right)\\ =\int_{\left[0,1\right)}\chi_{\;B}\left(x\right)d\left(\sigma_{k_{0}}\circ \sigma_{k_{1}}\circ \sigma_{k_{2}}\circ \cdots \circ \sigma_{k_{n}}\left(x\right)\right)\\ =\int_{\left[0,1\right)}\chi_{\;B}\left(x\right)\sigma_{k_{0}}^{\prime }\sigma_{k_{1}}^{\prime }\sigma_{k_{2}}^{\prime }\cdots \sigma_{k_{n}}^{\prime }\left(x\right)dx=2^{-n}\lambda \left(B\right)=\lambda \left(I_{n}\right)\lambda \left(B\right), \end{array}$$

that is $\lambda \left(I_{n}\right)\lambda \left(B\right)=\lambda \left(B\cap I_{n}\right)\leq C\lambda \left(B\cap I_{n}\right),$ where C=1. Thus, either the Lebesgue measure λ(B)=1 or λ(B)=0, so the doubling map (18) is ergodic.

**Example** **2.** *A very interesting example is given by the classical continued fraction expansion via the Gauss ergodic mapping*

(28) f:[0,1)∋x→{1/x}∈[0,1).

*whose fibering is defined by the mapping k:[0,1)∋x→⌊1/x⌋∈N:=D, with a generating partition of sets $X_{i}=\left(1/\left(i+1\right),1/i\right],i\in N,X={⊔}_{i\in N}X_{i}.$*

The probabilistic invariant measure is the well-known Gauss measure dμ(x)=dλ(x)/[(1+x)ln2], where dλ(x):=dx,x∈[0,1). The related entropy $h_{\mu }\left(f\right)$ of the Gauss mapping (28) is given by the Krengel–Rokhlin integral

(29) $$\begin{array}{cc} h_{\mu }\left(f\right) & =\frac{1}{\ln2}\int_{0}^{1}\frac{\ln|f^{\prime }\left(x\right)|}{1+x}dx=\frac{1}{\ln2}\int_{0}^{1}\frac{\ln x^{-2}}{1+x}dx=\frac{-2}{\ln2}\int_{0}^{1}\frac{\ln x}{1+x}dx=\\ \; & =\frac{-2}{\ln2}\int_{0}^{1}\left(\sum_{n\in Z_{+}}{\left(-1\right)}^{n}x^{n}\ln x\right)dx=\frac{1}{\ln2}\sum_{n\in Z_{+}}\frac{1}{n^{2}}=\frac{\pi^{2}}{6\ln2}, \end{array}$$

coinciding with the well-known [47,48,65] result. The ergodicity of the map (28) can be easily proved by reducing it [49] via the continued fraction expansion to a Bernoulli shift and applying Lemmas 1 and 2.

Namely, take a number x∈[0,1) and denote by $\left[x_{0},x_{1},\cdots ,x_{n},\cdots \right]$ its continuous fraction expansion:

(30) $$x=\frac{1}{x_{0}+}\frac{1}{x_{2}+\cdots }\frac{1}{x_{n}+\cdots },$$

where $x_{i}\in Z_{+}\;$ for all indices $i\in Z_{+}.$ Observe here that the induced continuous fraction mapping acts by left shifting as $T_{\varphi }\left[x_{0},x_{1},\cdots ,x_{n},\cdots \right]=\left[x_{1},\cdots ,x_{n},\cdots \right]$ for any expansion (30). This expansion $\left[x_{0},x_{1},\cdots ,x_{n},\cdots \right]$ can be reduced to *n*-th by defining for every t∈[0,1) the rational *t*-fraction

(31) $$\left[x_{0},x_{1},\cdots ,x_{n-1}+t\right]:=\frac{P_{n}\left(x_{0},x_{1},\cdots ,x_{n-1};t\right)}{Q_{n}\left(x_{0},x_{1},\cdots ,x_{n-1};t\right)},$$

where $P_{n}\left(x_{0},x_{1},\cdots ,x_{n-1};t\right)$ and $Q_{n}\left(x_{0},x_{1},\cdots ,x_{n-1};t\right)$ are coprime polynomials in the variables $x_{0},x_{1},\cdots ,x_{n-1}\in Z_{+}$ and t∈[0,1) for all n∈N. If we define the *n*-th order polynomials $P_{n}=P_{n}\left(x_{0},x_{1},\cdots ,x_{n-1}\right):=P_{n}\left(x_{0},x_{1},\cdots ,x_{n-1};0\right)$ and $Q_{n}=Q_{n}\left(x_{0},x_{1},\cdots ,x_{n-1}\right)$:=$Q_{n}\left(x_{0},x_{1},\cdots ,x_{n-1};0\right),$ it is easy to see that the following iterative expressions hold:

(32) $$\begin{array}{cc} P_{n}\left(x_{0},x_{1},\cdots ,x_{n-1};t\right) & =P_{n}+tP_{n-1},\\ Q_{n}\left(x_{0},x_{1},\cdots ,x_{n-1};t\right) & =Q_{n}+tQ_{n-1},\\ P_{n}\left(x_{0},x_{1},\cdots ,x_{n-1}\right) & =Q_{n-1}\left(x_{1},\cdots ,x_{n-1}\right),\\ P_{n+1}\left(x_{0},x_{1},\cdots ,x_{n-1},x_{n};t\right) & =x_{n}P_{n}+P_{n-1}+tP_{n},\\ Q_{n+1}\left(x_{0},x_{1},\cdots ,x_{n-1},x_{n};t\right) & =x_{n}Q_{n}+Q_{n-1}+tQ_{n}\; \end{array}$$

for any t∈[0,1) and arbitrary n∈N. By setting the parameter t=0 in (32), one readily obtains derives the following iterative relationships for all n∈N:

(33) $$P_{n+1}=x_{n}P_{n}+P_{n-1},Q_{n+1}=x_{n}Q_{n}+Q_{n-1}$$

with initial conditions $P_{0}=0,P_{1}=1$ and $Q_{0}=1,Q_{1}=x_{0}\in Z_{+}.$ In particular, the following invariant condition $Q_{n}$$P_{n-1}-$$P_{n}$$Q_{n-1}={\left(-1\right)}^{n}$ and inequality $Q_{n-1}\leq Q_{n\;\;}$ readily follow from (33) for all n∈N. Let $k_{0},k_{1},\cdots ,k_{n-1}\in N$ for every n∈N and define the cylindrical intervals $I_{n}$⊂[0,1) as the corresponding collection of rational *t*-fractions

(34) $$I_{n}=I_{n}\left(k_{0},k_{1},\cdots ,k_{n-1}\right):=\left\{\left[k_{0},k_{1},\cdots ,k_{n-1}+t\right]:t\in \left[0,1\right)\right\}.$$

Defining the inverse mappings $\;\left[0,1\right)∋x\to \frac{k}{k+x}\in I_{1}\left(k\right)$⊂[0,1),k∈N, one easily obtains the composition

(35) $$\sigma_{k_{0}}\circ \sigma_{k_{1}}\circ \cdots \circ \sigma_{k_{n-1}}:\left[0,1\right)\to I_{n}\left(k_{0},k_{1},\cdots ,k_{n-1}\right)\subset \left[0,1\right)$$

for every n∈N. Moreover, the condition $\varphi^{n}\circ \sigma_{k_{0}}\circ \sigma_{k_{1}}\circ \cdots \circ \sigma_{k_{n-1}}\left(x\right)=x$ holds for every x∈[0,1) and n∈N. Now we select any t∈[0,1), note that

(36) $$\sigma_{k_{0}}\circ \sigma_{k_{1}}\circ \cdots \circ \sigma_{k_{n-1}}\left(t\right)=\left[k_{0},k_{1},\cdots ,k_{n-1}+t\right]=\frac{P_{n}+tP_{n-1}}{Q_{n}+tQ_{n-1}},$$

and estimate the Lebesgue measure of the interval (34) to be

(37) $$\begin{array}{c} \lambda \left(I_{n}\right):=\int_{\left[0,1\right)}\chi_{I_{n}\left(t\right)dt}=\int_{I_{n}}dt=\int_{\left[0,1\right)}\left|J_{\sigma_{k_{0}}\circ \sigma_{k_{1}}\circ \cdots \circ \sigma_{k_{n-1}}}\left(t\right)\right|dt\\ =\int_{\left[0,1\right)}\left|\frac{d}{dt}\left(\frac{P_{n}+tP_{n-1}}{Q_{n}+tQ_{n-1}}\right)\right|dt=\int_{\left[0,1\right)}\frac{dt}{|Q_{n}+tQ_{n-1}{|}^{2}}\in \left[\frac{1}{4Q_{n}^{2}},\frac{1}{Q_{n}^{2}}\right], \end{array}$$

where we took into account that $0<Q_{n-1}\leq Q_{n\;\;}$ for all n∈N.

At this stage, we are in a position to estimate the Lebesgue measure $\lambda (B\cap I_{n})$ for the intersection $B\cap I_{n}$ of an invariant set $B=f^{-1}B=f^{-n}B\subset \left[0,1\right)$ and arbitrary cylindrical interval $I_{n}\subset \left[0,1\right),$n∈N as

(38) $$\begin{array}{c} \lambda \left(B\cap I_{n}\right)=\int_{I_{n}}\chi_{B}\left(x\right)dx\\ =\int_{\left[0,1\right)}\chi_{B}\left(\sigma_{k_{0}}\circ \sigma_{k_{1}}\circ \cdots \circ \sigma_{k_{n-1}}\left(x\right)\right)dx\\ =\int_{\left[0,1\right)}\chi_{\varphi^{-n}B}\left(\sigma_{k_{0}}\circ \sigma_{k_{1}}\circ \cdots \circ \sigma_{k_{n-1}}\left(x\right)\right)\left|J_{\sigma_{k_{0}}\circ \sigma_{k_{1}}\circ \cdots \circ \sigma_{k_{n-1}}}\left(x\right)\right|dx\\ =\int_{\left[0,1\right)}\chi_{B}\left(\varphi^{n}\circ \sigma_{k_{0}}\circ \sigma_{k_{1}}\circ \cdots \circ \sigma_{k_{n-1}}x\right)\left|J_{\sigma_{k_{0}}\circ \sigma_{k_{1}}\circ \cdots \circ \sigma_{k_{n-1}}}\left(x\right)\right|dx\\ =\int_{\left[0,1\right)}\chi_{B}\left(x\right)\left|J_{\sigma_{k_{0}}\circ \sigma_{k_{1}}\circ \cdots \circ \sigma_{k_{n-1}}}\left(x\right)\right|dx=\int_{\left[0,1\right)}\chi_{B}\left(x\right)\frac{dt}{|Q_{n}+xQ_{n-1}{|}^{2}}\\ \geq \frac{1}{4Q_{n}^{2}}\lambda \left(B\right)\geq \frac{1}{4}\lambda \left(I_{n}\right)\lambda \left(B\right), \end{array}$$

which is consistent with Lemma 2 with C=4. Consequently, from the estimate (38) one deduces that the measure λ(B)=1 or λ(B)=0, thus proving the ergodicity both of the Lebesgue measure dλ(x),x∈[0,1), and the invariant Gauss measure $d\mu \left(x\right)=\frac{dx}{(1+x)\ln2},x\in \left[0,1\right),$ on the unit interval [0,1).

## 3. One-Dimensional Boole-Type Mappings, Invariant Ergodic Measures and Their Entropies

The classical one-dimensional Boole [4] mapping is defined as

(39) β:R∖{0}∋x→x−1/x∈R.

Adler and Weiss in [46] proved that (39) is ergodic with respect to the infinite invariant σ-finite Lebesgue measure dλ(x):=dx,x∈R:=X. Their proof of the ergodicity relied heavily on the measure theoretic reduction of β to the corresponding *induced* [47,48,49] transformation $\beta_{A}:\left[-1,1\right]\to \left[-1,1\right]\subset R$ on the covering set A:=[−1,1], for which $R=\cup_{n\in N}\beta^{-n}\left(A\right)$ modulo a set of measure zero. The β-invariance of the Lebesgue measure dλ(x):=dx,x∈R, is easily checked making use of the Perron–Frobenius theory; namely, for preimages $u_{\pm }:=$$u_{\pm }\left(x\right)\in R,x$∈R, where $\beta (u_{\pm }\left(x\right))=x,$$u_{+}+$ $u_{-}=x,$$u_{-}$$u_{+}=-1,$ it is straightforward to show that

(40) $$\begin{array}{c} \sum_{\pm }du_{\pm }\left(x\right)=\sum_{\pm }\left|\frac{du_{\pm }}{dx}\right|dx=\sum_{\pm }\frac{dx}{|J_{B}\left(u_{\pm }\right)|}=\sum_{\pm }\frac{dx}{\left(1+u_{\pm }^{-2}\right)}=\\ =\sum_{\pm }\frac{u_{\pm }^{2}dx}{\left(1+u_{\pm }^{2}\right)}=\frac{(u_{+}^{2}+2\;+u_{-}^{2})dx}{1+{\left(u_{+}u_{-}\right)}^{2}+u_{+}^{2}+u_{-}^{2}}=\frac{\;(u_{+}^{2}+2+u_{-}^{2})dx}{2+u_{+}^{2}+u_{-}^{2}}=dx, \end{array}$$

coinciding exactly with the Lebesgue measure on R.

**Remark** **1.** *Here we remark that the Boole transformation (39) is σ-finite and nonsingular on R; that is, for any $A\subset R,\mu (\beta^{-1}A)=0$ iff μ(A)=0. Moreover, for nonsingular transformations f:X→X the following properties [1] are equivalent: (i) f is conservative and ergodic; (ii) for every set A⊂X,μ(A)>0 implies $\mu (X\setminus \cup_{n\in N}f^{-n}A)=0;$ (iii) for every set A⊂X,μ(A)>0 implies that for a.e. x∈X there exists an integer $n_{A}>0,$ such that $f^{n_{A}}\left(x\right)$∈A; (iv) for all sets A,B⊂X,μ(A)μ(B)>0 implies the existence of an integer n>0, such that $\mu (f^{-n}A\cap B)>$0; v) any A ⊂X,$f^{-1}\left(A\right)\subset A$ implies that $\mu \left(A\right)=0\vee \mu \left(\bar{A}\right)=0,$ where $\bar{A}:=X\setminus A,$ the complementary set to A∈B.*

We now consider a limiting version of the corresponding distributed Krengel type entropy of the Boole mapping (39) with respect to the σ-finite set of probabilistic measures $d\mu^{\left(r\right)}:=dx/\left(2r\right)$ on compact intervals [−r,r]⊂R as r→∞, which unfortunately can readily be shown to yield

(41) $$h_{\mu }\left(\beta \right)=\lim_{r\to \infty }\frac{1}{2r}\int_{-r}^{r}\ln\left(1+x^{-2}\right)dx=\lim_{r\to \infty }\frac{1}{r}\int_{0}^{r}\ln\left(1+x^{-2}\right)dx=\lim_{r\to \infty }\frac{1}{r}I\left(r\right)=0,$$

since I(r) is bounded on (0,∞). Not only is this entropy result counterintuitive, it is not actually valid since we cannot use the Krengel–Rokhlin formula (5) directly to calculate the entropy of maps of σ-finite measure preserving dynamical systems on spaces of infinite measure. Consequently, we propose to use a result from [11] for approximate Boole transformations that satisfy the necessary conditions for the validity of (5), which enables the calculation of the desired result as a limit. To this end, we define

(42) $$\beta_{\alpha }:R\setminus \left\{0\right\}\to R,\;$$

where $\beta_{\alpha }\left(x\right):=\alpha x-\left(1/x\right)$ and 0<α<1. The following (differential) probability measure on R, is absolutely continuous with respect to the (differential) Lebesgue measure dx and $\beta_{\alpha }$-invariant for all α∈(0,1):

(43) $$d\mu_{\alpha }\left(x\right):=\frac{\sqrt{\left(1-\alpha \right)}dx}{\pi [x^{2}\left(1-\alpha \right)+1]}$$

It is worth mentioning that the measure (43), suitably de-regularized, weakly tends to the Lebesgue measure dλ=dx on R; that is, *w*-$\lim_{\alpha ↑1}\frac{\pi d\mu_{\alpha }}{\sqrt{\left(1-\alpha \right)}}=d\lambda$ subject to the space $C_{0}\left(R;R\right)$. Now we can use the invariant probability measure (43) in the Krengel–Rokhlin formula (5) to obtain the following result in the limit:

(44) $$\begin{array}{cc} h_{\mu }\left(\beta \right) & :=\lim_{\alpha ↑1}h_{\mu_{\alpha }}\left(\beta_{\alpha }\right)=\lim_{\alpha ↑1}\;\int_{R}\ln\;\left(\frac{\left(\alpha x^{2}+1\right)\left[\left(1-\alpha \right)x^{2}+1\right]}{\left(1+\alpha -\alpha^{2}\right)x^{2}+\left(\alpha -1\right)}\right)\frac{\sqrt{\left(1-\alpha \right)}dx}{\pi [x^{2}\left(1-\alpha \right)+1]}\\ \; & ={\left.\frac{2}{\pi }\int_{0}^{\infty }\ln\left(1+x^{-2}\right)\frac{dx}{1+x^{2}}\right|}_{x=\tan s}=\frac{4}{\pi }\int_{0}^{\pi /2}\ln\left(1/\sin s\right)ds, \end{array}$$

so that

(45) $$\frac{4}{\pi }\int_{0}^{\pi /2}\ln\left(\frac{1}{s}\right)ds\leq h_{\mu }\left(\beta \right)\leq \frac{4}{\pi }\int_{0}^{\pi /2}\ln\left(\frac{\pi }{2s}\right)ds,$$

and

(46) $$\ln2<\frac{4}{\pi }\int_{0}^{\pi /2}\ln\left(\frac{1}{s}\right)ds≃1.09\leq h_{\mu }\left(\beta \right)\leq 2=\frac{4}{\pi }\int_{0}^{\pi /2}\ln\left(\frac{\pi }{2s}\right)ds.$$

Therefore, inasmuch as isomorphic measurable dynamical systems have the same entropy, (46) implies, for example, that β is not isomorphic to the doubling map (18). Additionally, it should be mentioned that the metric entropy of (42) can also be obtained using Pesin’s formula whenever α is positive and α≠1 (cf. [66]).

Admittedly, one might well question the limit-based computation (44) of the entropy. In particular, it is not rigorous because the very first equality, due to the fact entropy is only upper semi-continuous, is really just a lower bound. Therefore, we now use a compactification procedure to both confirm the result and also determine the topological entropy, which, as is well known [37], bounds the measure-theoretic entropy. We start by using what is essentially 1-dimensional stereographic projection and smooth extension to obtain a representation of the Boole map on the unit circle $S^{1}:=\left\{\left(X,Y\right)\in R^{2}:X^{2}+Y^{2}=1\right\}$ in the X,Y-plane via the commutative diagram

(47) $$\begin{array}{ccc} \;\;\;\;\;R\setminus \{0\} & \;\overset{\beta }{\;⟶}\; & R\\ \;\varphi ↓\;\;\; & \; & \;↓\varphi \\ \;S^{1} & \;\overset{\tilde{\beta }}{⟶}\; & S^{1}\subset R^{2} \end{array}$$

where

(48) $$\varphi \left(x\right):=\left(\frac{2x}{x^{2}+1},\frac{x^{2}-1}{x^{2}+1}\right)=\left(X\left(x\right),Y\left(x\right)\right)=\left(\cos\theta ,\sin\theta \right),\;\;\varphi^{-1}\left(X,Y\right)=\frac{Y+1}{X},$$

and

(49) $$\begin{array}{cc} \tilde{\beta }\left(X,Y\right) & :=\varphi \circ \beta \circ \varphi^{-1}\left(X,Y\right)={\left(1+3Y^{2}\right)}^{-1}\left(4XY,5Y^{2}-1\right)\\ \; & ={\left(1+3\sin^{2}s\right)}^{-1}\left(2\sin2\theta \;,5\sin^{2}s-1\right). \end{array}$$

Observe that $\tilde{\beta }$ is a smooth (= $C^{\infty }$) surjection of the unit circle onto itself, having among others the properties that there is a unique fixed point at the north pole, (0,1) corresponding to θ=s=π/2 and $\tilde{\beta }\left(1,0\right)=\tilde{\beta }\left(-1,0\right)=\left(0,-1\right)$, so ${\tilde{\beta }}^{2}\left(1,0\right)={\tilde{\beta }}^{2}\left(-1,0\right)=\tilde{\beta }\left(0,-1\right)=\left(0,1\right)$. It is also worth noting that the differential of arclength ds for which $d\tilde{\mu }:=$ds/2π is a natural (differential) probability measure on $S^{1}$ satisfies

(50) $$ds:=\sqrt{dX^{2}+dY^{2}}=\frac{2dx}{x^{2}+1}.$$

It is straightforward to show from its definition that probability measure $\tilde{\mu }$ on $S^{1}$ is absolutely continuous with respect to the Lebesgue measure associated to the representation of the unit circle as R/Z and that it is $\tilde{\beta }$-invariant. Moreover, a simple calculation reveals that $\tilde{\beta }$ is a dilation $\tilde{\mu }$-almost everywhere on $S^{1}$; in fact, $\frac{d}{ds}\tilde{\beta }\left(s\right)>1$ except at the fixed point (0,1). Consequently, the Krengel–Rokhlin formula can be used to compute the entropy, so that owing to the change of variables formula for integrals and (44), we find that

(51) $$h_{\tilde{\mu }}\left(\tilde{\beta }\right)=\frac{1}{2\pi }\int_{S^{1}}\ln\left(\frac{d}{ds}\tilde{\beta }\left(s\right)\right)ds=\frac{1}{\pi }\int_{R}\ln\left(1+x^{-2}\right)\frac{dx}{1+x^{2}}=\frac{4}{\pi }\int_{0}^{\pi /2}\ln\left(1/\sin u\right)du.$$

Serendipitously, this formula for the K-S entropy of the measurable dynamical system $\left(S^{1},M,\tilde{\mu },\tilde{\beta }\right)$ can also be used to obtain the formula for the topological entropy of the topological dynamical system $\left(S^{1},T,\tilde{\beta }\right)$, where M is the (Borel) $\tilde{\mu }$-measurable subsets of the unit circle and T is the Euclidean subspace topology on $S^{1}$. In fact, it is not difficult to verify that the hypotheses of Theorem 3 of [35] are satisfied for $\left(S^{1},M,\tilde{\mu },\tilde{\beta }\right)$ and $\left(S^{1},T,\tilde{\beta }\right)$, so it follows that

(52) $$h_{T}\left(\tilde{\beta }\right)=h_{\hat{\mu }}\left(\tilde{\beta }\right)=\frac{4}{\pi }\int_{0}^{\pi /2}\ln\left(1/\sin u\right)du.$$

Next, we present a new proof of the ergodicity of the Boole transformation (39)—a variant of the approach in [59]—that is more concise than the original due to Adler and Weiss [46].

**Theorem** **3.** *The one-dimensional Boole transformation (39) is ergodic with respect to the invariant Lebesgue measure λ on R.*

**Proof.**  As mentioned above, our argument can be reduced to Theorem 2, by using the relation of (54) to the doubling mapping T:[0,1)∋ s→{2s}∈[0,1). As was also shown in [14,26,60], the Boole transformation (39) is related to the doubling mapping T:[0,1)∋s→{2s}∈[0,1) via the commutative diagram

(53) $$\begin{array}{ccccc} \left[0,1\right) & \overset{\cot(\pi \circ )}{\to } & R & \overset{\beta }{\to } & R\\ ↓↑Id & \; & & \; & ↓\pi^{-1}\cot^{-1}\\ & \overset{T}{\to } & \left[0,1\right) & \overset{\alpha^{-1}\left(\pi \circ \right)}{\to } & \left[0,1\right) \end{array},$$

where $\alpha^{-1}:\left[0,1\right)\to \left[0,1\right)\;$ is a diffeomorphism, defined by $\alpha \left(s\right):=\;\;\pi^{-1}\left[\pi /2+\tan^{-1}\left(\pi s/2\right)\right],$s∈[0,1), related to the map (39) as

(54) $$\;\beta =\cot\pi \circ \alpha^{-1}\circ T\left(\pi^{-1}\right)\circ \cot^{-1}.$$

Now $\hat{\beta }$$:\left[0,1\right)\to \left[0,1\right),\hat{\beta }$ $:=\alpha^{-1}\left(\pi \right)\circ T,$ is equivalent to (54), where

(55) $$\hat{\beta }\left(s\right):=\pi^{-1}\cot^{-1}\left(\beta \left(\cot\left(\pi s\right)\right)\right)$$

for any s∈[0,1). As every a∈[0,1) has the binary expansion

(56) $$a:=\left(\cdot k_{0}k_{1}k_{2}\cdots k_{n}\cdots \right)=\sum_{j\in Z_{+}}k_{j}2^{-(j+1)},$$

one can define the so-called proper [14,60] cylindrical sets $\;I_{n}:=I_{n}\left(k_{0},k_{1,}\cdots ,k_{n}\right)\subset \left[0,1\right),n\in Z_{+},$ as

(57) $$I_{n}=\left\{\left(\sigma_{k_{n-1}}\circ \alpha \right)\circ \left(\sigma_{k_{n-1}}\circ \alpha \right)\circ \cdots \circ \left(\sigma_{k_{0}}\circ \alpha \right)\left(t\right):t\in \left[0,1\right)\right\}$$

where $\sigma_{0}\left(s\right)=s/2,$ if s∈[0,1/2), and $\;\sigma_{1}\left(s\right)=\left(1+s\right)/2,$ if s∈[1/2,1). Note also that $\hat{\beta }\circ \left(\sigma_{k_{j}}\circ \alpha \right)\left(s\right)=s$ for every s∈[0,1),$k_{j}\in \left\{0,1\right\},j=\bar{0,n-1}.$ The Lebesgue measure of the interval (57) can be easily estimated as follows:

(58) $$\begin{array}{cc} \lambda (I_{n}) & =\int_{I_{n}}dx={\left.\int_{R}\chi_{I_{n}}\left(x\right)dx\right|}_{x=\cot(\pi t)}\\ \; & =\int_{\left[0,1\right)}\left|J_{\left(\sigma_{k_{n}}\circ \alpha \right)\circ \left(\sigma_{k_{n-1}}\circ \alpha \right)\circ \cdots \circ \left(\sigma_{k_{0}}\circ \alpha \right))}\left(t\right)\right|dt\\ \; & =\int_{\left[0,1\right)}\left(\sigma_{k_{n-1}}^{\prime }\alpha^{\prime }\left(t_{n-1}\right)\right)\left(\sigma_{k_{n-1}}^{\prime }\alpha^{\prime }\left(t_{n-1}\right)\right)\cdots \left(\sigma_{k_{0}}^{\prime }\alpha^{\prime }\left(t\right)\right)dt, \end{array}$$

where the derivatives $\sigma_{k_{j}}^{\prime }=1/2,\;\alpha^{\prime }\left(t_{j}\right)=2/\left[1+3\sin^{2}\left(\pi t_{j}\right)\right],$$t_{j}:=\sigma_{k_{j}}\circ \alpha \circ \cdots \circ \sigma_{k_{0}}\circ \alpha \left(t\right),j=\bar{0,n-1},t\in \left[0,1\right).$ Since

(59) $$\begin{array}{c} \lambda \left(\sigma_{0}\circ \alpha \right)\left(\left[0,1\right)\right])=1/2=\lambda (\sigma_{1}\circ \alpha \left(\left[0,1\right]\right),\\ \sigma_{k_{j}}\circ \alpha \circ \cdots \circ \sigma_{k_{0}}\circ \alpha \left(\left[0,1\right)\right)\subset \left[1/2^{j+1},1/2^{j}\right) \end{array}$$

for any $j=\bar{0,n-1},$ owing to the classical mean value theorem applied to (59), one easily finds that

(60) $$\begin{array}{c} \alpha^{\prime }\left({\bar{t}}_{j}\right))\;=2^{-j}/\left[3\sin^{2}\left(\pi {\bar{t}}_{j}\right)+1\right], \end{array}$$

where ${\bar{t}}_{j}\in \left(1/2^{j+1},1/2^{j}\right)\subset \left[0,1\right)$ for all $j\in \bar{0,n}.$ Hence, owing to the evident inequalities 2t≤sin(πt)≤πt for all t∈[0,1/2), one derives the estimates

(61) $$\begin{array}{cc} \;\frac{2^{-j}}{\exp[3{\left(\pi 2^{-j}\right)}^{2}} & \leq \frac{2^{-j}}{3\sin^{2}\left(\pi 2^{-j}\right)+1}\leq \frac{2^{-j}\;}{3\sin^{2}\left(\pi t_{j}\right)+1}\leq \\ \; & \leq \frac{2^{-j}}{3\sin^{2}\left(\pi 2^{-(j+1)}\right)+1}\leq \frac{2^{-j}}{3{\left(2^{-(j+1)}\right)}^{2}+1} \end{array}$$

for any $j=\bar{0,n}$. Thus, from expressions (58) and (61) one immediately deduces the required Renyi-type estimates [14,47,48,49,63,67,68,69].

(62) $$\begin{array}{c} \frac{\exp(-3\pi^{2})}{2^{n(n+1)/2}}\leq \frac{\exp[\;\pi^{2}\left(-4+4^{-n}\right)]}{2^{n(n+1)/2}}=\prod_{j=0}^{n}\frac{2^{-j}}{\exp[3{\left(\pi 2^{-j}\right)}^{2}}\leq \lambda \left(I_{n}\right)\leq \\ \leq \prod_{j=0}^{n}\frac{2^{-j}}{3{\left(2^{-(j+1)}\right)}^{2}+1}\leq \frac{2^{-n(n+1)/2}}{\sum_{j=0}^{n}3{\left(2^{-(j+1)}\right)}^{2}+1}=\frac{2^{-n(n+1)/2}}{2-4^{-(n+1)}}\leq \frac{4/7}{2^{n(n+1)/2}} \end{array}$$

for all $n\in Z_{+}.$ In particular, it follows from (62) that $\lim_{n\to \infty }\lambda \left(I_{n}\right)=0,$ meaning that the family of these cylindrical sets generates [26,60,64] the Borel σ-algebra B on the interval [0,1). Thus, we are now in a position to apply Lemmas 1 and 2. So, let a measurable set B⊂[0,1) be invariant; i.e., $B=\beta^{-1}B=\beta^{-n}B,n\in N,$ and calculate the following Lebesgue measure:

(63) $$\begin{array}{c} \lambda \left(B\cap I_{n}\right)=\int_{\left[0,1\right)}\chi_{B\cap I_{n}}\left(t\right)dt=\int_{\left[0,1\right)}\chi_{B}\left(t\right)\chi_{I_{n}}\left(t\right)dt=\int_{I_{n}}\chi_{B}\left(t\right)dt\\ =\int_{\left[0,1\right)}\chi_{B}\left(\left(\sigma_{k_{n}}\circ \alpha \;\right)\circ \left(\sigma_{k_{n-1}}\circ \alpha \right)\circ \cdots \circ \left(\sigma_{k_{0}}\circ \alpha \right)\left(t\right)\right)\times \\ \times d(\left(\sigma_{k_{n}}\circ \alpha \right)\circ \left(\sigma_{k_{n-1}}\circ \alpha \right)\circ \cdots \circ \left(\sigma_{k_{0}}\circ \alpha \right)\left(x\right))\\ =\int_{\left[0,1\right)}\chi_{\beta^{-n}B}\left(\left(\sigma_{k_{n}}\circ \alpha \right)\circ \left(\sigma_{k_{n-1}}\circ \alpha \right)\circ \cdots \circ \left(\sigma_{k_{0}}\circ \alpha \right)\left(x\right)\right)\times \\ \times d(\left(\sigma_{k_{n}}\circ \alpha \right)\circ \left(\sigma_{k_{n-1}}\circ \alpha \right)\circ \cdots \circ \left(\sigma_{k_{0}}\circ \alpha \right)\left(x\right))\\ =\int_{\left[0,1\right)}\chi_{B}\left({\hat{\beta }}^{n}\circ \left(\sigma_{k_{n}}\circ \alpha \right)\circ \left(\sigma_{k_{n-1}}\circ \alpha \right)\circ \cdots \circ \left(\sigma_{k_{0}}\circ \alpha \right)\left(t\right)\right)\times \\ \times d(\left(\sigma_{k_{n}}\circ \alpha \right)\circ \left(\sigma_{k_{n-1}}\circ \alpha \right)\circ \cdots \circ \left(\sigma_{k_{0}}\circ \alpha \right)\left(t\right)). \end{array}$$

Since the composition $\;\;\beta \circ (\sigma_{k_{j}}\circ \alpha )=Id$ for any $j=\bar{0,n},$ from (63) one deduces

$$\begin{array}{c} \lambda \left(B\cap I_{n}\right)=\int_{\left[0,1\right)}\chi_{\;B}\left(x\right)d\left(\left(\sigma_{k_{n}}\circ \alpha \right)\circ \left(\sigma_{k_{n-1}}\circ \alpha \right)\circ \cdots \circ \left(\sigma_{k_{0}}\circ \alpha \right)\left(x\right)\right)\\ =\int_{\left[0,1\right)}\chi_{\;B}\left(x\right)\sigma_{k_{n}}^{\prime }\alpha^{\prime }\sigma_{k_{n-1}}^{\prime }\alpha^{\prime }\sigma_{k_{n-2}}^{\prime }\alpha^{\prime }\cdots \sigma_{k_{0}}^{\prime }\alpha^{\prime }\left(x\right)dx\\ \geq \frac{\exp(-3\pi^{2})}{2^{n(n+1)/2}\lambda \left(I_{n}\right)}\lambda \left(I_{n}\right)\lambda \left(B\right)\geq \frac{7}{4\exp(3\pi^{2})}\lambda \left(I_{n}\right)\lambda \left(B\right); \end{array}$$

that is, the Lebesgue measure satisfies $\lambda \left(I_{n}\right)\lambda \left(B\right)\leq C\lambda \left(B\cap I_{n}\right)\;$ for all $n\in Z_{+},$ where $C=4\exp(3\pi^{2})/7.$ Thus, owing to Lemma 2, either λ(B)=1 or λ(B)=0, which proves the ergodicity of the Boole mapping (39) with respect to the same invariant Lebesgue measure λ on R and completes the proof. □

It is also worth mentioning here the well-known result [1,13,47,48,70,71] that the doubling map (18) is isomorphic to the 1-dimensional Boole-type transformation

(64) f:R∋x→(x−1/x)/2∈R,

which is invariant with respect to the probability measure $d\mu \left(x\right)=dx/[\pi \left(1+x^{2}\right)],$x∈R, and has entropy

(65) $$\begin{array}{c} h_{\mu }\left(f\right)=\frac{1}{\pi }\int_{R}\frac{\ln[\left(1+x^{-2}\right)/2]dx}{\left(1+x^{2}\right)}=\frac{2}{\pi }\int_{0}^{\infty }\frac{\ln[\left(1+x^{-2}\right)/2]dx}{\left(1+x^{2}\right)}\\ =\frac{2}{\pi }\int_{0}^{\infty }\frac{\ln(1+x^{-2})dx}{\left(1+x^{2}\right)}-\frac{2\ln2}{\pi }\int_{0}^{\infty }\frac{dx}{\left(1+x^{2}\right)}\\ =\frac{2}{\pi }\int_{0}^{\infty }\frac{\tan^{-1}x\;dx}{(1+x^{2})x}-\ln2=\frac{4}{\pi }\left(\frac{\pi }{2}\ln2\right)-\ln2=\ln2, \end{array}$$

coinciding with that of (19). The Boole mapping (39) was also generalized [1] in the form

(66) $$R\setminus \left\{b_{j}:j=\bar{1,N}\right\}∋x\to \tilde{f}\left(x\right):=\alpha x+a-\sum_{j=1}^{N}\frac{\beta_{j}}{x-b_{j}}\in R,$$

where *a*, $b_{j}\in R,$$j=\bar{1,N},$$\alpha ,\beta_{j}\in R_{+},$$j=\bar{1,N},$ and analyzed in [1,3,11,72,73]. For α=1,a=0, the ergodicity result was proved in [3,74,75,76] by making use of a specially devised inner function method. The related spectral aspects of the mapping (66) were in part also studied in [1,3]. In spite of these results, the case α≠1 is still challenging, with the only available related results [1,3] being for the special case of (66)

(67) $$R∋x\to f_{\#}\left(x\right):=\alpha x+a-\frac{\beta }{x-b}\in R$$

for α=1/2, and arbitrary a,b∈R and $\beta \in R_{+}.\;$ Invariant measures and ergodicity related to (67) were analyzed in [11,70,71,72] using their equivalence to

(68) [0,1)∋s:→T(s)=2smod1∈[0,1),

following from the commutative diagram

(69) $$\begin{array}{ccc} \left[0,1\right) & \overset{T}{\to } & \left[0,1\right)\\ \varphi ↓\; & \; & ↓\varphi \\ R\; & \overset{f_{\#}}{\to } & R \end{array},$$

for which the condition $\varphi \circ T=\varphi \circ f_{\#},$ where φ(s):= ${\left(2\beta \right)}^{1/2}\cot\left(\pi s\right)+2a,s\in \left[0,1\right),$ holds. The Krengel–Rokhlin formula (5) can be used to calculate the corresponding measure-theoretic entropy of the $f_{\#}$, yielding $\;h_{\mu }\left(f_{\#}\right)=\ln2,$ which is the entropy of the shift map (19).

It is also important to mention that the theory of inner functions in [1,74,75,76] was used to prove the existence an $f_{\#}$-invariant probability measure $\mu_{\#}$ on R, such that the generalized Boole type transformation (66) is ergodic for any N>1, α=1 and a=0. It appears that the transformation (66) is not ergodic for α=1 and a≠0 since it is totally dissipative, with wandering set $\Omega^{c}\left(f_{\#}\right):=$$\cup W(f_{\#})$ =R, where $W(f_{\#})\subset R$ are such that all $f_{\#}^{-n}\left(W\right),$${n\in Z}_{+},$ are disjoint. An analogous result can be proved [1] for the generalized Boole-type transformation

(70) $$R∋x\to g\left(x\right):=\alpha x+a+\int_{R}\frac{d\nu (s)}{s-x}\in R,$$

where a∈R,$\alpha \in R_{+}$ and the measure ν on R (not necessary absolutely continuous with respect Lebesgue measure) has compact support suppν⊂R and satisfies the natural conditions

(71) $$\int_{R}\frac{d\nu (s)}{1+s^{2}}=a,\;\int_{R}d\nu \left(s\right)<\infty ,\;$$

which guarantee the boundedness of its entropy.

## 4. Multi-Dimensional Boole Transformations: Their Entropy and Ergodicity

Multi-dimensional endomorphisms of measurable spaces are of great interest [14,47] in many mathematical subdisciplines, including number theory, numerics, dynamical systems theory and diverse physical applications. In this regard, we should mention [14,61,62], which treat many interesting measure preserving and ergodic multi-dimensional mappings. Recently, in [11,70,72,73] a class of new multi-dimensional Boole type transformations $\beta_{\rho }:R^{n}\to R^{n}$ of the following form were introduced and analyzed

(72) $$\beta_{\rho }\left(x_{1},x_{2},\cdots ,x_{n}\right):=\left(x_{1}-1/x_{\rho (1)},x_{2}\pm 1/x_{\rho (2)},\cdots ,x_{n}\pm 1/x_{\rho (n)}\right)\;,\;$$

for any n∈N and arbitrary permutations ρ$\in S_{n},$ where the signs "±" are chosen from the nondegeneracy condition $J_{\beta_{\rho }}\left(x\right)\neq 0,x\in R^{n}\setminus \left\{0\right\}.$ For the case n=2,(x,y)$\in R^{2}\setminus \left\{0,0\right\},$ one obtains the two-dimensional Boole type mapping:

(73) $$\;\beta_{\left(21\right)}\left(x,y\right):=\left(x-1/y,y+1/x\right),\;\;$$

and for the case n=3,(x,y,z)$\in R^{3}\setminus \left\{0,0,0\right\},$ the pair of nontrivial three-dimensional Boole type mapping:

(74) $$\begin{array}{cc} \beta_{\left(231\right)}\left(x,y,z\right) & :=(x-1/y,y+1/z,z+1/x),\\ \beta_{\left(23_{-}1_{-}\right)}\left(x,y,z\right) & :=(x-1/y,y-1/z,z-1/x).\; \end{array}$$

The infinitesimal σ-finite Lebesgue measure dλ(x,y):=dxdy, $\left(x,y\right)\in R^{2}$ is $\beta_{\left(21\right)}$- invariant, as can be easily checked via the Perron–Frobenius eigenfunction condition as follows: For the corresponding preimages $(u_{\pm },v_{\pm }):=$$\left(u_{\pm }\left(x,y\right),v_{\pm }\left(x,y\right)\right)$$\in R^{2},$ where $u_{+}u_{-}=xy^{-1},v_{+}v_{-}=-yx^{-1},u_{+}+u_{-}=2y^{-1}+x,$$v_{+}+v_{-}=y-2x^{-1},$$\beta_{\left(21\right)}\left(u_{\pm },v_{\pm }\right)=\left(x,y\right)\in R^{2},$ one verifies that the measure satisfies

(75) $$\begin{array}{c} \sum_{\pm }du_{\pm }dv_{\pm }\left(x,y\right)=\sum_{\pm }\left|J_{\left(u_{\pm },v_{\pm }\right)}\left(x,y\right)\right|dxdy\\ =\sum_{\pm }\frac{dxdy}{|J_{F}\left(u_{\pm },v_{\pm }\right)|}=\sum_{\pm }\frac{dxdy}{\left(1+{\left(u_{\pm }v_{\pm }\right)}^{-2}\right)}\\ =\sum_{\pm }\frac{{\left(u_{\pm }v_{\pm }\right)}^{2}dx}{\left(1+{\left(u_{\pm }v_{\pm }\right)}^{2}\right)}=\frac{\left[2{\left(u_{+}v_{+}u_{-}v_{-}\right)}^{2}\;+{\left(u_{-}v_{-}\right)}^{2}+{\left(u_{+}v_{+}\right)}^{2}\right]dxdy}{\left[1+{\left(u_{-}v_{-}\right)}^{2}+{\left(u_{+}v_{+}\right)}^{2}+{\left(u_{+}v_{+}u_{-}v_{-}\right)}^{2}\right]}\\ =\frac{\;\left[{\left(u_{-}v_{-}\right)}^{2}+{\left(u_{+}v_{+}\right)}^{2}+2\right]dxdy}{\left[2+{\left(u_{-}v_{-}\right)}^{2}+{\left(u_{+}v_{+}\right)}^{2}\right]}=dxdy, \end{array}$$

coinciding with the Lebesgue measure dλ(x,y):=dxdy. As for the ergodicity of the Lebesgue measure preserving mapping (73), the approach based on Theorem 2 employing *smooth-fibered* multi-dimensional mappings does not seem to be viable. Inasmuch as that the ergodicity result of [46] for the one-dimensional Boole mapping (39) is largely based on the induced Kakutani transformation technique, one can expect that it is also applicable to the two-dimensional Boole map (72).

Now we recall that the notion of the induced transformation [47,48,49] for an infinite measure preserving mapping f:X→X, which was used by Adler and Weiss [46] to prove the ergodicity of the map (39), is rather closely related to the classical Poincaré recurrence theorem [18,48]. Namely, let (X;B,μ,f) be a measure preserving discrete dynamical system, where A⊂X is a set of positive measure satisfying the covering condition

(76) $$X=\cup_{n\in N}f^{-n}A\;$$

modulo a set of measure zero.

**Remark** **2.** *It is worth mentioning here [18,47,48,49] that if a measurable dynamical system (X;B,μ,f) satisfies for arbitrarily chosen measurable A⊂X,μ(A)>0, the covering condition (76), then f:X→X is ergodic. In fact, if f is ergodic, then for an arbitrary measurable set B⊂X,$\mu (B\Delta f^{-1}B)=0$ implies that μ(B)=1, or μ(B)=0. Now let A⊂X be a measurable set with μ(A)>0 and define $B:=\cup_{n\in N}f^{-n}A.$ Since $f^{-1}B\subset B,$ one infers that $\mu \left(f^{-1}B\right)=\mu \left(B\right),$ implying that $\mu (B\Delta f^{-1}B)=0,$ so μ(B)=1, or μ(B)=0. Moreover, as $f^{-1}A\subset B\;\;$ one concludes that μ(B)≥μ(A), or μ(B)=1. Whence, $B=\cup_{n\in N}f^{-n}A=X$ modulo a set of measure zero.*

*In general, for a probability measure space (X;B,μ) the following properties are equivalent: (i) f:X→X is ergodic; (ii) for any $A\in B,\mu (f^{-1}\left(A\right)△A)=0$ implies μ(A)=1 or μ(A)=0; (iii) for A∈B,μ(A)>0 implies that $\mu (\cup_{n\in N}f^{-n}\left(A\right))=1;$ iv) for A,B∈B,μ(A)μ(B)>0 implies that there exists $n_{A}\in N,$ such that $\mu (f^{-n_{A}}\left(A\right)\cap B)>0;$ (v) for any measurable and invariant function g:X→C,g=g∘fμ-almost everywhere implies that g:X→C is constant almost everywhere.*

Owing to the condition (76), the first return time $\tau_{A}\in N\;\;$ can now be defined as

(77) $$\tau_{A}\left(x\right):=\underset{n\in N}{\inf}\left\{n:f^{n}\left(x\right)\in A,x\in A\right\},$$

which exists almost everywhere and is finite.

**Definition** **1.** *Let a measurable dynamical system (X;B,μ,f) satisfy the condition (76). Then a mapping $f_{A}:A\to A$ defined as*

(78) $$f_{A}\left(x\right):=f^{\tau_{A}\left(x\right)}\left(x\right)$$

*for almost all x∈A is called the transformation induced by f:X→X on the subset A⊂X.*

The induced transformation is characterized by the following [47,48,49] important theorems:

**Theorem** **4.** *(M.Kac). Let a mapping f:X→X be ergodic and a measurable set A⊂X be such that 0<μ(A)<∞. Then the average return time is proportional to the measure μ(A); that is,*

(79) $$\int_{A}\tau_{A}\left(x\right)d\mu \left(x\right)=\mu \left(A\right).$$

**Theorem** **5.** *The induced transformation (78) is measure preserving on the space $(A,B{|}_{A},\mu_{A}=\mu {\left(A\right)}^{-1}\mu {|}_{A},f_{A}),$ where ${B|}_{A}:=\left\{B\cap A:B\in B\right\},0<\mu \left(A\right)<\infty .$ Moreover, if f:X→X is ergodic, with respect to the measure μ, the induced transformation $f_{A}:A\to A$ is ergodic with respect to the measure $\mu_{A}:=\mu /\mu \left(A\right)$ induced on the set A.*

An instructive proof of this theorem is given below in Supplement to the article. As was already mentioned above, this theorem was used in [46] to prove the ergodicity of the Boole mapping (39). In addition, it was shown above that there is a second effective essentially analytical approach to proving the ergodicity, and so it would be useful to present two proofs, if any, of the ergodicity of the two-dimensional Boole type mapping (73).

Concerning the approach based on Theorem 5, its main technical ingredients are intimately related to the construction of a special generating partition of the measure space X, suggested by Kakutani and Rokhlin [51,77] for the corresponding induced mapping $f_{A}:A\to A$.

Now, if one tries to apply the measure-theoretic construction devised in [46] for proving ergodicity of the two-dimensional Boole mapping (73), some very technical difficulties arise that appear to be too difficult to overcome. Thus, the analytical approach based on Theorem 2 that relies on the relationship between the two-dimensional Boole mapping (73) and the two-dimensional doubling map appears to be the only feasible choice. More specifically (73), which for convenience in what follows we define as F:=$\beta_{\left(21\right)}$, is related to the following two-dimensional transformation $\;T_{F}:{\left[0,1\right)}^{2}∋\left(s,t\right)\to \left(\left\{2s\right\},\left\{2t\right\}\right)\in {\left[0,1\right)}^{2}$ on the square $Y={\left[0,1\right)}^{2}\subset R^{2},$ according to the commutative diagram

(80) $$\begin{array}{ccccc} {\left[0,1\right)}^{2} & \overset{\cot(\pi \circ )}{\to } & R^{2} & \overset{F}{\to } & R^{2}\\ S↓ & \; & & \; & ↓\cot^{-1}\pi \\ {}^{2} & \overset{T_{F}\left(\circ \right)}{\to } & \;{\left[0,1\right)}^{2} & \overset{\alpha^{-1}}{\to } & {\left[0,1\right)}^{2} \end{array},$$

where $\alpha^{-1}:{\left[0,1\right)}^{2}\to R^{2}$ is the map

(81) $$\alpha^{-1}\left(\begin{array}{c} s\;\\ t \end{array}\right):=\left(\begin{array}{c} \alpha_{1}^{-1}\left(s,t\right)\;\\ \alpha_{2}^{-1}\left(s,t\right) \end{array}\right)=\left(\begin{array}{c} \;\;\pi^{-1}\cot^{-1}\left(\frac{2\cot\{\pi (s+t)\}}{1+\sin\{\pi (s-t)\}/\sin\{\pi (s+t)\}}\right)\\ \;\pi^{-1}\cot^{-1}\left(\frac{2\cot\{\pi (s-t)\}}{-1+\sin\{\pi (s+t)\}/\sin\{\pi (s-t)\}}\right) \end{array}\right)\;$$

owing to changing the variables x=cot(πs),y=cot(πt), $\left(s,t\right)\in {\left[0,1\right)}^{2},\;\left(x,y\right)\in R^{2},$ subject to the new coordinates $\left(s,t\right)\in {\left[0,1\right)}^{2}$ and the transformation $S^{-1}:{\left[0,1\right)}^{2}∋\left(s,t\right)\to$$\left(\left\{s+t\right\},\left\{s-t\right\}\right)\in {\left[0,1\right)}^{2}.$ That this approach could be used to prove of the ergodicity theorem of the two-dimensional Boole transformation (73), was announced in [70,71,72] and is now confirmed by the following result.

**Theorem** **6.** *The two-dimensional Boole transformation $F=\beta_{\left(21\right)}$ defined in (73) is ergodic with respect to the invariant Lebesgue measure λ on $R^{2}.$*

**Proof.**  We begin by constructing the proper cylindrical subsets $I_{n}:=I_{n}(k_{0},k_{1,}\cdots ,k_{n-1};$$l_{0},l_{1,}\cdots ,l_{n-1}{)\subset \left[0,1\right)}^{2},\;$$n\in Z_{+}:$

(82) $$I_{n}=\;\left\{\overset{⟵}{\prod_{j=\bar{0,n-1}}}\left(S^{-1}\circ \sigma_{k_{j},l_{j}}\circ \alpha \right):\left(u,v\right)\in {\left[0,1\right)}^{2}\right\}\;$$

for the diffeomorphically equivalent $\tilde{F}$-mapping $\tilde{F}={\left({\tilde{F}}_{1},{\tilde{F}}_{2}\right)}^{⊺}:{\left[0,1\right)}^{2}\to {\left[0,1\right)}^{2},$ where $\;T_{F}\circ \sigma_{k_{j},l_{j}}=Id:{\left[0,1\right)}^{2}\to {\left[0,1\right)}^{2},\;\sigma_{k_{j},l_{j}}:={\left(\sigma_{k_{j}},\sigma_{l_{j}}\right)}^{-1},k_{j},l_{j}\in \left\{0,1\right\},j=\bar{0,n-1},\sigma_{0}\left(s\right)=s/2,$ if s∈[0,1/2),$\;\sigma_{1}\left(s\right)=\left(1+s\right)/2,$ if s∈[1/2,1) and

(83) $${\left({\tilde{F}}_{1},{\tilde{F}}_{2}\right)}^{⊺}=\cot^{-1}\left(\pi \circ \right)\alpha^{-1}\circ T_{F}\circ S,$$

satisfies the obvious conditions ${\tilde{F}}_{1}\circ \;\left(S^{-1}\circ \pi^{-1}\cot^{-1}\;\circ \pi \sigma_{k_{j}}\circ \alpha_{1}\circ \cot\left(\pi \circ \right)\right)\left(u,v\right)=u,$ ${\tilde{F}}_{2}\circ \;\left(S^{-1}\circ \pi^{-1}\cot^{-1}\;\circ \pi \sigma_{k_{j}}\circ \alpha_{1}\circ \cot\left(\pi \circ \right)\right)\left(u,v\right)=v$ for every $\left(u,v\right)\in {\left[0,1\right)}^{2},$$k_{j},l_{j}\in \bar{0,1},j\in \bar{0,n-1}.\;$ Now the Lebesgue measure of the cylindrical interval (82) can be now easily estimated as follows. We have the Lebesgue interval measure

(84) $$\begin{array}{cc} \lambda (I_{n}) & =\int_{I_{n}}dudv=\int_{{\left[0,1\right)}^{2}}\chi_{I_{n}}\left(u,v\right)dudv\\ \; & =\int_{{\left[0,1\right)}^{2}}\left|J_{\left(S^{-1}\circ \sigma_{k_{n-1},l_{n-1}}\circ \alpha \right)\circ \left(S^{-1}\circ \sigma_{k_{n-2},l_{n-2}}\circ \alpha \right)\circ \cdots \circ \left(S^{-1}\circ \sigma_{k_{0},l_{0}}\circ \alpha \right)}\left(u,v\right)\right|dudv\\ \; & =\int_{{\left[0,1\right)}^{2}}\prod_{j=\bar{0,n-1}}|J_{S^{-1}}|J_{\sigma_{k_{j},l_{j}}}|J_{\alpha }\left(u_{j},v_{j}\right)\left|dudv=\frac{1}{4^{n}}\int_{{\left[0,1\right)}^{2}}\prod_{j=\bar{0,n-1}}J_{\alpha }\left(u_{j},v_{j}\right)\right|dudv \end{array}$$

where $S^{-1}\circ \sigma_{k_{j},l_{j}}\circ \alpha \left(u_{j},v_{j}\right):=\left(u_{j+1},v_{j+1}\right)\in {\left[2^{-(j+1)},2^{-j}\right)}^{2},\tilde{\varphi }\left(u_{j+1},v_{j+1}\right)=\left(u_{j},v_{j}\right),$$j=\bar{0,n-1},\left(u_{0},v_{0}\right)$$:=\left(u,v\right)\in {\left[0,1\right)}^{2}.$ Taking into account that

(85) $$\;\left(S^{-1}\circ \sigma_{k_{j-1},l_{j-1}}\circ \alpha \right)\circ \left(S^{-1}\circ \sigma_{k_{n-2},l_{n-2}}\circ \alpha \right)\circ \cdots \circ \left(S^{-1}\circ \sigma_{k_{0},l_{0}}\circ \alpha \right)\left({\left[0,1\right)}^{2}\right)\subset \left[1/2^{j+1},1/2^{j}\right)\;$$

and 2t≤sinπt≤πt,2s≤sinπs≤πs for all s,t∈[0,1/2], the subintegral Jacobian product of (84) can be represented as

(86) $$\begin{array}{c} \prod_{j=\bar{0,n-1}}J_{\alpha }\left(u_{j},v_{j}\right)=\prod_{j=\bar{0,n-1}}\frac{\left[\cos^{2}\pi \left(u_{j}+v_{j}\right)+\sin^{2}\pi u_{j}\cos^{2}\pi v_{j}\right]\left[\cos^{2}\pi \left(u_{j}-v_{j}\right)+\sin^{2}\pi v_{j}\cos^{2}\pi u_{j}\right]}{\left(1-\sin^{2}\pi u_{j}-\sin^{2}\pi v_{j}+2\sin^{2}\pi u_{j}\sin^{2}\pi v_{j}\right)}=\\ =\prod_{j=\bar{0,n-1}}\frac{\left[1-\sin^{2}\pi v_{j}+\sin^{2}\pi u_{j}\sin^{2}\pi v_{j}-1/2\sin^{2}2\pi u_{j}\sin2\pi v_{j}\right]\left[1-\sin^{2}\pi u_{j}+\sin^{2}\pi u_{j}\sin^{2}\pi v_{j}+1/2\sin^{2}2\pi u_{j}\sin2\pi v_{j}\right]}{\left(1-\sin^{2}\pi u_{j}-\sin^{2}\pi v_{j}+2\sin^{2}\pi u_{j}\sin^{2}\pi v_{j}\right)}. \end{array}$$

This can be readily estimated as

(87) $$\begin{array}{c} \left(\frac{3\pi^{2}}{4}+\frac{1}{4^{2}}-1\right)\left(\frac{\pi^{2}}{4}-\frac{3}{2}+\frac{1}{4^{2}}\right)\exp\left[\sum_{j\in N}\left(\frac{1-\pi^{2}}{4^{j}}+\frac{2(1-\pi^{4})}{16^{j}}\right)\right]\\ \leq \prod_{j=\bar{0,n-1}}J_{\alpha }\left(u_{j},v_{j}\right)\leq \;{\left[1-\frac{\pi^{2}}{2}+{\left(\frac{\pi }{2}\right)}^{4}\right]}^{-1}\exp\left[\sum_{j\in N}\left(\frac{2\pi^{2}+6}{4^{j+1}}+\frac{1}{16^{j+1}}\right)\right]. \end{array}$$

Thus, based on the estimates (86), we readily obtain the following inequalities for the measure (84):

(88) $$\;\frac{C_{1}}{4^{n}}\leq \lambda \left(I_{n}\right)\leq \frac{C_{2}}{4^{n}}$$

for any n∈N, where the constants are

(89) $$\begin{array}{cc} C_{1} & :=\left(\frac{3\pi^{2}}{4}+\frac{1}{4^{2}}-1\right)\left(\frac{\pi^{2}}{4}-\frac{3}{2}+\frac{1}{4^{2}}\right)\exp\left[\sum_{j\in N}\left(\frac{1-\pi^{2}}{4^{j}}+\frac{2(1-\pi^{4})}{16^{j}}\right)\right],\\ C_{2} & :={\left[1-\frac{\pi^{2}}{2}+{\left(\frac{\pi }{2}\right)}^{4}\right]}^{-1}\exp\left[\sum_{j\in N}\left(\frac{2\pi^{2}+6}{4^{j+1}}+\frac{1}{16^{j+1}}\right)\right]. \end{array}$$

Having the estimate (88), we are in a position allowing to apply Lemmas 1 and 2. Whence, if a measurable set $B\subset {\left[0,1\right)}^{2}$ is *F*-invariant so that $B=F^{-1}B=F^{-n}B,n\in N,$ we compute that

(90) $$\begin{array}{c} \lambda \left(B\cap I_{n}\right)=\int_{{\left[0,1\right)}^{2}}\chi_{B\cap I_{n}}\left(u,v\right)dudv=\int_{{\left[0,1\right)}^{2}}\chi_{B}\left(u,v\right)\chi_{I_{n}}\left(u,v\right)dudv=\int_{I_{n}}\chi_{B}\left(u,v\right)dudv\\ =\int_{{\left[0,1\right)}^{2}}\chi_{B}\left(\left(S^{-1}\circ \sigma_{k_{n-1},l_{n-1}}\circ \alpha \;\right)\circ \left(S^{-1}\circ \sigma_{k_{n-2},l_{n-2}}\circ \alpha \right)\circ \cdots \circ \left(S^{-1}\circ \sigma_{k_{0},l_{0}}\circ \alpha \right)\left(t\right)\right)\times \\ \times d\lambda (\left(S^{-1}\circ \sigma_{k_{n-1},l_{n-1}}\circ \alpha \;\right)\circ \left(S^{-1}\circ \sigma_{k_{n-2},l_{n-2}}\circ \alpha \right)\circ \cdots \circ \left(S^{-1}\circ \sigma_{k_{0},l_{0}}\circ \alpha \right)\left(u,v\right))\\ =\int_{\left[0,1\right)}\chi_{F^{-n}B}\left(\left(S^{-1}\circ \sigma_{k_{n-1},l_{n-1}}\circ \alpha \;\right)\circ \left(S^{-1}\circ \sigma_{k_{n-2},l_{n-2}}\circ \alpha \right)\circ \cdots \circ \left(S^{-1}\circ \sigma_{k_{0},l_{0}}\circ \alpha \right)\right)\left(u,v\right))\times \\ \times d\lambda \left(\left(S^{-1}\circ \sigma_{k_{n-1},l_{n-1}}\circ \alpha \;\right)\circ \left(S^{-1}\circ \sigma_{k_{n-2},l_{n-2}}\circ \alpha \right)\circ \cdots \circ \left(S^{-1}\circ \sigma_{k_{0},l_{0}}\circ \alpha \right)\right)\left(u,v\right))\\ =\int_{\left[0,1\right)}\chi_{B}\left({\tilde{F}}^{n}\circ \left(S^{-1}\circ \sigma_{k_{n-1},l_{n-1}}\circ \alpha \;\right)\circ \left(S^{-1}\circ \sigma_{k_{n-2},l_{n-2}}\circ \alpha \right)\circ \cdots \circ \left(S^{-1}\circ \sigma_{k_{0},l_{0}}\circ \alpha \right)\left(u,v\right)\right)\times \\ \times \times d\lambda \left(\left(S^{-1}\circ \sigma_{k_{n-1},l_{n-1}}\circ \alpha \;\right)\circ \left(S^{-1}\circ \sigma_{k_{n-2},l_{n-2}}\circ \alpha \right)\circ \cdots \circ \left(S^{-1}\circ \sigma_{k_{0},l_{0}}\circ \alpha \right)\right)\left(u,v\right))\\ =\int_{{\left[0,1\right)}^{2}}\chi_{\;B}\left(u,v\right)\left|J_{\left(S^{-1}\circ \sigma_{k_{n-1},l_{n-1}}\circ \alpha \right)\circ \left(S^{-1}\circ \sigma_{k_{n-2},l_{n-2}}\circ \alpha \right)\circ \cdots \circ \left(S^{-1}\circ \sigma_{k_{0},l_{0}}\circ \alpha \right)}\left(u,v\right)\right|dudv\\ =\int_{{\left[0,1\right)}^{2}}\chi_{\;B}\left(u,v\right)\prod_{j=\bar{0,n-1}}|J_{S^{-1}}|J_{\sigma_{k_{j},l_{j}}}\left|J_{\alpha }\left(u_{j},v_{j}\right)\right|dudv=\frac{1}{4^{n}}\int_{B}\;\prod_{j=\bar{0,n-1}}J_{\alpha }\left(u_{j},v_{j}\right)dudv, \end{array}$$

where we made use of the property that the composition $\;F\circ (S^{-1}\circ \sigma_{k_{j},l_{j}}\circ \alpha )=Id$ for any $j=\bar{0,n-1}.$ Now, it follows from (88) that

$$\begin{array}{c} \lambda \left(B\cap I_{n}\right)=\frac{1}{4^{n}}\int_{{\left[0,1\right)}^{2}}\chi_{\;B}\left(u,v\right)\prod_{j=\bar{0,n-1}}J_{\alpha }\left(u_{j},v_{j}\right)dudv\\ \geq \frac{C_{1}}{4^{n}\lambda \left(I_{n}\right)}\;\lambda \left(I_{n}\right)\lambda \left(B\right)\geq C_{1}C_{2}^{-1}\lambda \left(I_{n}\right)\lambda \left(B\right), \end{array}$$

so the Lebesgue measure satisfies $\lambda \left(I_{n}\right)\lambda \left(B\right)\leq C\lambda \left(B\cap I_{n}\right)$ for all $n\in Z_{+},$ where $C:=C_{2}C_{1}^{-1}.$ Therefore, owing to Lemma 2, either λ(B)=1 or λ(B)=0, thus confirming the ergodicity of the two-dimensional Boole mapping (73) with respect to the same invariant Lebesgue measure λ on $R^{2},$ and completing the proof. □

As mentioned above, the Lebesgue measure on $R^{3}$ is invariant with respect to the three-dimensional Boole-type transformations (74), which are also likely to be ergodic, but the search for a proof is still ongoing.

## 5. Supplement: Proof of Theorem 6

Let f:X→X be ergodic and consider a measurable set A⊂X satisfying the condition 0<μ(A)<∞ together with its induced mapping $f_{A}:A\to A.$ As the condition (76) *a priori* [18,46,47,48,49] holds, one can construct the following disjoint measurable *first return iteration* subsets

(91) $$X_{n}:=\left\{x\in X:f^{n}\left(x\right)\in A,f^{j}\left(x\right)\notin A,j=\bar{1,n-1}\right\},$$

where ${⊔}_{n\in N}X_{n}=X,X_{n}\cap X_{m}=⌀,$m≠n∈N, and for which

(92) $$X_{n+1}=f^{-1}X_{n}\cap f^{-1}\bar{A}$$

is satisfied. Using the sets (91), one constructs for all n∈N the sets

(93) $$A_{n}:=X_{n}\cap A,\;\;\;B_{n}:=X_{n}\cap \bar{A},$$

satisfying the disjoint sum property

(94) $$\begin{array}{cc} f^{-1}B_{n} & =B_{n+1}⊔A_{n+1},\\ {⊔}_{n\in N}A_{n} & =A,{⊔}_{n\in N}B_{n}=\bar{A}. \end{array}$$

For any measurable subset E⊂A we note that

(95) $$f_{A}^{-1}E={⊔}_{n\in N}\left(f^{-n}E\cap A_{n}\right),$$

which gives rise to the equality

(96) $$\mu \left(f_{A}^{-1}E\right)=\mu \left({⊔}_{n\in N}\left(f^{-n}E\cap A_{n}\right)\right)=\sum_{n\in N}\mu \left(f^{-n}E\cap A_{n}\right).$$

Employing the representation (94) and the measure invariance, one readily deduces that

(97) $$\begin{array}{c} \mu \left(E\right)=\mu \left(f^{-1}E\right)=\mu \left(f^{-1}E\cap \left(B_{1}⊔A_{1}\right)\right)\\ =\mu \left(f^{-1}E\cap B_{1}\right)+\mu \left(f^{-1}E\cap A_{1}\right),\\ \mu \left(B_{n}\right)=\mu \left(f^{-1}B_{n}\right)=\mu \left(B_{n+1}⊔A_{n+2}\right)=\mu \left(B_{n+1}\right)+\mu \left(A_{n+2}\right),\\ \mu \left(f^{-1}E\cap B_{1}\right)=\mu \left(f^{-1}\left(f^{-1}E\cap B_{1}\right)\right)=\mu \left(f^{-2}E\cap f^{-1}B_{1}\right)\\ =\mu \left(f^{-2}E\cap \left(B_{2}⊔A_{2}\right)\right)=\mu \left(f^{-2}E\cap B_{2}\right)+\mu \left(f^{-2}E\cap B_{2}\right),\\ ...\\ \mu \left(f^{-n}E\cap B_{n}\right)=\mu \left(f^{-(n+1)}E\cap B_{n+1}\right)+\mu \left(f^{-(n+1)}E\cap A_{n+1}\right), \end{array}$$

for all n∈N. Consequently, we find that

(98) $$\mu \left(f^{-n}E\cap B_{n}\right)=\sum_{k=n+1}^{\infty }\mu \left(f^{-n}E\cap A_{n}\right),\mu \left(B_{n}\right)=\sum_{k=n+1}^{\infty }\mu \left(A_{n}\right),$$

which reduces to

(99) $$\begin{array}{c} \mu \left(f^{-n}E\cap B_{n}\right)+\sum_{k=\bar{1,n}}\mu \left(f^{-n}E\cap A_{n}\right)\\ =\sum_{n\in N}\mu \left(f^{-n}E\cap A_{n}\right):=\eta_{A},\;\\ \mu \left(A\right)=\sum_{k=1}^{\infty }\mu \left(A_{n}\right),\;\;\;\mu \left(B_{1}\right)=\sum_{k=2}^{\infty }\mu \left(A_{n}\right). \end{array}$$

Whence follows the invariance of the positive quantity $\eta_{A}\in R_{+}$ with respect to n∈N and the boundedness of the measure $\mu \left(B_{1}\right)\leq \mu \left(A\right),$ since the measure μ(A)=$\mu ({⊔}_{n\in N}A_{n})<\infty \;$ is assumed bounded. It follows immediately from the first equality of (97) that $\eta_{A}=\mu \left(A\right)>0,$ that is

(100) $$\mu \left(f^{-n}E\cap B_{n}\right)+\sum_{k=\bar{1,n}}\mu \left(f^{-n}E\cap A_{n}\right)=\mu \left(E\right).$$

In light of (96), it follows from (100) and (99) that

(101) $$\begin{array}{c} |\mu \left(f_{A}^{-1}E\right)-\mu \left(E\right)|=\lim_{n\to \infty }\left(\sum_{k=n+1}^{\infty }\mu \left(f^{-k}E\cap A_{k}\right)\right.\\ +\mu \left(f^{-n}E\cap B_{n}\right))\leq 2\lim_{n\to \infty }\left(\sum_{k=n+1}^{\infty }\mu \left(\;A_{k}\right)\right)=0 \end{array}$$

owing to the convergence condition (99) for the measure μ(A)<∞, so $\mu \left(f_{A}^{-1}E\right)=\mu \left(E\right)$ for any measurable set E⊂A. Therefore, the suitably induced measure on A⊂X, $\mu_{A}=\mu /\mu \left(A\right)$, is also invariant with respect to the induced map $f_{A}:A\to A.$

If we assume now that the induced mapping $f_{A}:A\to A$ is ergodic and take an *f*-invariant set *D*⊂X such that μ(D∩A)>0, then since either μ(D∩A)>0 or $\mu (D\cap \bar{A})>0,$ it follows from the expansion (95) that

(102) $$\begin{array}{c} f_{A}^{-1}\left(D\cap A\right)={⊔}_{n\in N}\left(f^{-n}\left(D\cap A\right)\cap A_{n}\right)\\ ={⊔}_{n\in N}\left(D\cap f^{-n}A\cap A_{n}\right)=D\cap \left({⊔}_{n\in N}\left(f^{-n}A\cap A_{n}\right)\right)\\ =D\cap f_{A}^{-1}A=D\cap A, \end{array}$$

since the initial assumption $\cup_{n\in N}f^{-n}A=X$ guarantees that $f_{A}^{-1}A=A$ modulo a set of measure zero. As the induced mapping is assumed to be ergodic, from (102) and the condition μ(D∩A)>0, we immediately conclude that D∩A=A. Thus, based once more on the initial assumption $\cup_{n\in N}f^{-n}A=X$ one readily finds that

(103) $$\begin{array}{cc} X & =\cup_{n\in N}f^{-n}\left(D\cap A\right)=\cup_{n\in N}\left(f^{-n}D\cap f^{-n}A\right)\\ \; & =\cup_{n\in N}\left(D\cap f^{-n}A\right)=D\cap \left(\cup_{n\in N}f^{-n}A\right)=D\cap X=D, \end{array}$$

meaning that the mapping f:X→X is also ergodic.

Similarly, one can also prove the converse statement. In particular, if the mapping f:X→X is ergodic and a set E⊂A of positive measure is $f_{A}$-invariant, then

(104) $$f_{A}^{-1}E={⊔}_{n\in N}\left(f^{-n}E\cap A_{n}\right)=E.$$

Taking into account the invariance condition (104), let us construct the set $G:=E\cup {⊔}_{n\in N}\left(B_{n}\cap f^{-n}E\right)$ and calculate its inverse image under *f* as follows:

(105) $$\begin{array}{c} f^{-1}G=f^{-1}E\cup {⊔}_{n\in N}\left(f^{-1}B_{n}\cap f^{-(n+1)}E\right)\\ =f^{-1}E\cup {⊔}_{n\in N}\left(\left(B_{n+1}⊔A_{n+1}\right)\cap f^{-(n+1)}E)\right)\\ =\;\;f^{-1}E\cup \;\;\left({⊔}_{n\in N}\left(B_{n+1}\cap f^{-(n+1)}E\right)\right)⋃\\ ⋃f^{-1}E\cup \;\;\left({⊔}_{n\in N}\left(A_{n+1}\cap f^{-(n+1)}E\right)\right)\\ =\left(A_{1}\cap f^{-1}E⊔B_{1}\cap f^{-1}E\right)\cup \;\;\left({⊔}_{n\in N}\left(B_{n+1}\cap f^{-(n+1)}E\right)\right)⋃\\ ⋃\;\left(A_{1}\cap f^{-1}E⊔B_{1}\cap f^{-1}E\right)\cup \;\left({⊔}_{n\in N}\left(A_{n+1}\cap f^{-(n+1)}E\right)\right)\\ =\;\left({⊔}_{n\in N}\left(B_{n}\cap f^{-n}E\right)\right)⋃\left({⊔}_{n\in N}\left(A_{n}\cap f^{-n}E\right)\right)\\ =\left({⊔}_{n\in N}\left(B_{n}\cap f^{-n}E\right)\right)\cup E=G. \end{array}$$

Hence, *G* is *f*-invariant, so the ergodicity of *f* implies that G=X modulo a set of measure zero. Having now taken into account that, by construction, the subset ${⊔}_{n\in N}\left(B_{n}\cap f^{-n}E\right)$ $\subset \bar{A},$ it follows that the set A⊆E modulo a set of measure zero in X. Consequently, from the assumption that E⊂A, one deduces finally that A=E, which means that the induced mapping $f_{A}:A\to A$ is also ergodic, proving the theorem.

## 6. Conclusions

We delineated several aspects of discrete measurable and differential dynamical systems essential to our investigation of the classical Boole transformation and some of its generalizations and extensions. The aspects of primary interest were metric and topological entropy and ergodicity and their interrelationships. For example, the Rokhlin–Krengel formula for metric entropy of ergodic systems played a key role in our treatment of both topological and Kolmogorov–Sinai entropy. One of the main results obtained was the calculation of the topological and metric entropy of the classical (one-dimensional) Boole map employing a limiting approach as well as a compactification method based on stereographic projection. In addition, we presented a new proof of the ergodicity of the classical Boole map employing the ideas of Li and Schweiger and studied the entropy of some simple generalizations of the Boole map.

An interesting class of multi-dimensional extensions of the classical Boole map was introduced and the members were shown to be invariant under the Lebesgue measures of the corresponding dimension. It was proved, using an approach based on fibered mappings, that a particular two-dimensional member of the class is ergodic and a similar result for the higher dimensional generalizations was conjectured. Moreover, we conjectured that the techniques used to compute both topological and metric entropy for the one-dimensional Boole map could be adapted for these multi-dimensional extensions as well. As for related future research, we plan to investigate both our ergodicity and entropy conjectures in considerable detail.

## Data Availability

Not applicable.
